# Peripheral and central arterial pressure and its relationship to vascular target organ damage in carotid artery, retina and arterial stiffness. Development and validation of a tool. The Vaso risk study

**DOI:** 10.1186/1471-2458-11-266

**Published:** 2011-04-27

**Authors:** Luis Garcia-Ortiz, Emilio Ramos-Delgado, Jose I Recio-Rodriguez, Cristina Agudo-Conde, Carlos Martínez-Salgado, Maria C Patino-Alonso, Emiliano Rodriguez-Sanchez, Manuel A Gomez-Marcos

**Affiliations:** 1 Unidad de Investigación de Atención Primaria La Alamedilla, Salamanca, Spain; 2 Unidad de Fisiopatología Renal y Cardiovascular, Instituto Reina Sofía de Investigación Nefrológica, Universidad de Salamanca, Salamanca, Spain

## Abstract

**Background:**

Ambulatory blood pressure monitoring (ABPM) shows a better correlation to target organ damage and cardiovascular morbidity-mortality than office blood pressure. A loss of arterial elasticity and an increase in carotid artery intima-media thickness (IMT) has been associated with increased cardiovascular morbidity-mortality. Tools have been developed that allow estimation of the retinal arteriovenous index but not all studies coincide and there are contradictory results in relation to the evolution of the arteriosclerotic lesions and the caliber of the retinal vessels. The purpose of this study is to analyze the relationship between peripheral and central arterial pressure (clinic and ambulatory) and vascular structure and function as evaluated by the carotid artery intima-media thickness, retina arteriovenous index, pulse wave velocity (PWV) and ankle-brachial index in patients with and without type 2 diabetes. In turn, software is developed and validated for measuring retinal vessel thickness and automatically estimating the arteriovenous index.

**Methods/Design:**

A cross-sectional study involving a control group will be made, with a posterior 4-year follow-up period in primary care. The study patients will be type 2 diabetics, with a control group of non-diabetic individuals. Consecutive sampling will be used to include 300 patients between 34-75 years of age and no previous cardiovascular disease, one-half being assigned to each group. Main measurements: age, gender, height, weight and abdominal circumference. Lipids, creatinine, microalbuminuria, blood glucose, HbA1c, blood insulin, high sensitivity C-reactive protein and endothelial dysfunction markers. Clinic and ambulatory blood pressure monitoring. Carotid ultrasound to evaluate IMT, and retinography to evaluate the arteriovenous index. ECG to assess left ventricle hypertrophy, ankle-brachial index, and pulse wave analysis (PWA) and pulse wave velocity (PWV) with the Sphigmocor System.

**Discussion:**

We hope to obtain information on the correlation of different ABPM-derived parameters and PWA to organ target damage - particularly vascular structure and function evaluated from the IMT and PWV - and endothelial dysfunction in patients with and without type 2 diabetes. We also hope to demonstrate the usefulness of the instrument developed for the automated evaluation of retinal vascularization in the early detection of alterations in vascular structure and function and in the prognosis of middle-term cardiovascular morbidity.

**Trial Registration:**

Clinical Trials.gov Identifier: NCT01325064

## Background

Blood pressure measurement at the clinic remains the standard of reference, but there are increasing evidence that the values obtained after home blood pressure measurement by the patient and particularly 24-hour ambulatory blood pressure monitoring (ABPM) [1] shows a better correlation to target organ damage and cardiovascular morbidity-mortality [2-4]. The prognostic value of resting blood pressure, particularly when the latter decreases, is greater than that of blood pressure during activity [5,6]. On the other hand, the ambulatory arterial stiffness index (AASI) [7] is a better estimator of central arterial stiffness than other classical indicators such as pulse pressure (PP) [8], and has been shown to be a useful predictor of vascular mortality, particularly in fatal and nonfatal stroke [9,10].

A loss of arterial elasticity, or arterial stiffness, has been associated with increased cardiovascular morbidity-mortality. At present the gold standard for evaluating arterial stiffness is carotid-femoral pulse wave velocity [11]. This parameter has been related to increased morbidity-mortality in both patients with cardiovascular disease and in healthy subjects [12,13]. It has also been observed that central arterial pressure is more strongly correlated to cardiovascular morbidity-mortality than peripheral arterial pressure [14]. In the ASCOT study [15], increased morbidity and mortality was found in one of the study groups compared to the other, despite the fact that the peripheral arterial pressures were similar in both groups. In the CAFÉ sub-study [16] the subjects with increased morbidity-mortality were found to have greater central arterial pressure and a higher augmentation index (AIx)(defined as the ratio between the increase in central systolic pressure and central pulse pressure) than the other group. The serum levels of osteoprotegerin (OPG) are elevated in patients with type 1 and 2 diabetes, representing an early marker contributing to the endothelial dysfunction associated with the disease. In addition, OPG concentration in plasma is correlated to the severity of peripheral arterial disease [17]. An increase in carotid artery intima-media thickness (IMT) is an early phenomenon in the development of arteriosclerosis. A number of studies in asymptomatic individuals have shown carotid IMT to be an independent risk factor for coronary disease and stroke [18-20].

The classical Keith Wagener classification applied to the analysis of retinal vascular lesions has some limitations, particularly in correctly evaluating the initial phases of the vascular lesions. Tools have been developed that allow estimation of the retinal arteriovenous index [18,20-23], and this index and arterial and venous caliber independently have been correlated to an increased risk of arterial hypertension, diabetes mellitus and cardio and cerebrovascular diseases [24-27]. However, not all studies coincide, and there are contradictory results in relation to the evolution of the arteriosclerotic lesions and the caliber of the retinal vessels. In addition, the existing tools are manual or semiautomated, and observer influence consequently can prove important. The development of automated tools designed to improve performance and lessen interobserver variability therefore appears necessary.

In a phase prior to this project we developed a tool that determines retinal artery and vein thickness and estimates the retina arteriovenous index based on a semiautomated method, with the purpose of obtaining an automatic tool for this study. Associated cardiovascular disorders are the main cause of mortality in diabetic patients [28]. Patients with diabetes mellitus show premature vascular aging compared with the non-diabetic population. This increases their vascular risk two- to three-fold in men and four- to five-fold in women, according to the European cardiovascular prevention guide of 2007, compared with the population without diabetes [29]. This makes it extremely important to ensure the early detection of arterial stiffness in this population, in order to adopt the opportune measures to lessen cardiovascular risk. Lastly, the correlation of central arterial pressure, the augmentation index (AI), pulse wave velocity (PWV) and AASI to target organ damage in diabetics has not been explored in depth. Likewise, the correlation to the retina arteriovenous index has not been specifically established to date. It is also necessary to better establish the prognostic value of central arterial pressure and PWV in relation to the evolution of the target organ damage markers and to the possible incidence of cardiovascular events in patients with type 2 diabetes.

The present project was therefore designed to analyze the relationship between arterial pressure (clinic and ambulatory), the arterial pressure circadian profile and other parameters generated by ABPM and central arterial pressure, and vascular structure and function as evaluated by the carotid IMT, retina arteriovenous index, PWV and ankle-brachial index (ABI) in patients with and without type 2 diabetes.

An evaluation also will be made of the prognostic usefulness of the retina arteriovenous index in relation to the presence of vascular damage and cardiovascular disease, and to biological markers of endothelial dysfunction. To this effect an automated tool will be developed for estimating retinal artery and vein calibers, and for calculating the retina arteriovenous index.

## Methods/Design

### Study design

The first phase will comprise a cross-sectional observational study with a control group, while the second phase will consist of a prospective observational study with annual follow-up during four years. The study will be carried out in the urban primary care setting.

### Subjects

The study population will be composed of type 2 diabetics, and an age- and gender-matched control group without diabetes will also be included. Based on consecutive sampling of all patients referred to the research unit for cardiovascular risk assessment, we will invite those who meet the inclusion criteria and show no reasons for exclusion to participate in the study, until the estimated sample sizes of both groups have been reached.

Inclusion criteria: Patients aged 34 years or older and less than 75 years, with and without type 2 diabetes. Exclusion criteria: patients unable to comply with the protocol requirements (psychological and/or cognitive disorders, failure to cooperate, educational limitations and problems for understanding), patients with ischemic heart disease, cerebrovascular disease or other atherosclerotic disease, patients participating or who will participate in a clinical trial during the study, and patients with serious comorbidities representing a threat to life over the subsequent 12 months.

The sample size was estimated to detect differences between the subjects with and without diabetes in reference to PWV, as this represents the most unfavorable situation. Accepting an alpha risk of 0.05 and a beta risk of 0.20 in two-sided contrasts, 149 subjects are required in the first group and 149 in the second, in order to detect a difference of ≥ 1 unit. The common standard deviation (SD) is taken to be 3 [30], with a potential 5% rate of losses or difficulties in the technique. This sample suffices to detect a difference of 0.01 units in the retina arteriovenous index, considering a SD of 0.028 [23] and losses due to difficulties in the technique of 10%. Accepting an alpha risk of 0.05 and a beta risk of 0.20 in two-sided contrasts, 137 subjects are required in the first group and 137 in the second. Thus, we aim to include 300 patients: one-half diabetics and the other half without diabetes.

### Variables and measurement instruments

#### Demographic and clinical variables

Age and gender. Family history of cardiovascular disease. Associated cardiovascular diseases, arterial hypertension, dyslipidemia, diabetes mellitus. History of smoking, alcohol consumption and physical activity. Arterial pressure and heart rate in clinic and with ABPM. Height, weight, and abdominal (waist) circumference. Blood sampling will be carried out under fasting conditions, and first morning urine will be collected. A blood sample and the urine will be sent to the core laboratory, and another blood sample will be centrifuged and frozen for posterior analysis. Initial determination will be made of blood glucose, HbA1c, insulinemia, microalbuminuria, blood and urine creatinine, lipids and high sensitivity C-reactive protein. Posteriorly, biological markers of endothelial dysfunction will be determined (endoglin and osteoprotegerin, OPG).

**The office blood pressure and heart rate **measurement will be obtained by performing three measurements of systolic blood pressure (SBP) and diastolic blood pressure (DBP), with a validated sphygmomanometer, OMRON M7 model (Omron Health Care, Kyoto, Japan), following the recommendations of the European Society of Hypertension [31]. The mean of the last two measurements obtained from the arm with high blood pressure by the nurse of the research unit will be used for the study.

**The Ambulatory blood pressure monitoring (ABPM) and heart rate **will be performed on a day of standard activity, with an adequate cuff for the size of the patient's arm. A SpaceLabs 90207 control system (Spacelabs Healthcare, Issaquah, Washington, USA), validated according to the protocol of the British Hypertension Society, was used [32]. The records of readings considered to be valid will be ≥ 66% of the total. Furthermore, for the records to be evaluable, at least 14 measurements were required during the daytime period, or at least 7 during the nighttime or rest period. The monitor will be programed for obtaining blood pressure measurements every 20 min. during the daytime period and every 30 min. during the rest period.

**Ambulatory arterial stiffness index (AASI) **will be defined as 1 minus the regression slope of DBP over SBP readings obtained from individual 24-hour blood pressure recordings The stiffer the arterial tree will be the closer the regression slope and AASI will be to 0 and 1, respectively [33].

**Pulse wave velocity (PWV) and Peripheral (PAIx) and central (CAIx) Augmentation Index **will be estimated through the SphymgoCor System (AtCor Medical Pty Ltd Head Office, West Ryde, Australia). Using the SphygmoCor System (Px Pulse Wave Analysis) by an investigator, with the patient in the sitting position and resting the arm on a rigid surface, pulse wave analysis will be made with a sensor in the radial artery, using mathematical transformation to estimate the aortic pulse wave. The reliability of which was evaluated before the study began using the intra-class correlation coefficient showed values of 0.977 (95%CI: 0.942 to 0.991) for inter-observer agreement and 0.979 (95%CI: 0.948 to 0.992) for intra-observer agreement on repeated measurements in 20 subjects, and according to the Bland-Altman analysis the limit of intra-observer agreement was 0.650 (-6.496 to 7.796) and inter-observer agreement was 1.250 (-5.914 to 8.414). From the morphology of the aortic wave, Central AIx will be estimated using the following formula: Increase in central pressure * 100/pulse pressure. Peripheral AIx will be calculated as follows: (Second peak systolic blood pressure [SBP2]-diastolic blood pressure [DBP])/(first peak SBP-DBP)×100(%). Using the SphygmoCor System (Vx Pulse Wave Velocity), and with the patient in the supine position, the pulse wave of the carotid and femoral arteries will be analyzed, estimating the delay with respect to the ECG wave and calculating the PWV. Distance measurements will be taken with a measuring tape from the sternal notch to the carotid and femoral arteries at the sensor location.

#### Assessment of carotid intima-media thickness (IMT)

Carotid ultrasonography to assess IMT will be performed by two investigators trained for this before starting the study. The reliability of which was evaluated before the study began using the intraclass correlation coefficient, which showed values of 0.974 (95%CI: 0.935 to 0.990) for intra-observer agreement on repeated measurements in 20 subjects, and 0.897 (95%CI:0.740 to 0.959) for inter-observer agreement, and according to the Bland-Altman analysis, the limit of inter-observer agreement was 0.022 (-0.053 to 0.098) and the limit of intra-observer agreement was 0.012 (-0.034 to 0.059). A Sonosite Micromax ultrasound device paired with a 5-10 Mhz multifrequency high-resolution linear transducer with Sonocal software will be used for performing automatic measurements of IMT in order to optimize reproducibility. Measurements will be made of the common carotid after the examination of a longitudinal section of 10 mm at a distance of 1 cm from the bifurcation, performing measurements in the anterior or proximal wall, and in the posterior or distal wall in the lateral, anterior and posterior projections, following an axis perpendicular to the artery to discriminate two lines, one for the intima-blood interface and the other to the media-adventitious interface. A total of 6 measurements will be obtained of the right carotid and other 6 of the left carotid, using average values (average IMT) and maximum values (maximum IMT) calculated by the software automatically. The measurements will be obtained with the subject lying down, with the head extended and slightly turned opposite to the carotid examined, following the recommendations of the Manheim Carotid Intima-Media Thickness Consensus [34].

#### Evaluation of peripheral artery involvement

This will be evaluated using the ankle-brachial index (ABI), performed in the morning without having consumed coffee or tobacco for at least 8 hours prior to measuring and an ambient temperature of 22-24°C. With the feet uncovered, in a supine decubitus position after 20 minutes of rest, the pressure in the lower extremities and blood pressure in both arms will be measured using a portable WatchBP Office ABI (Microlife AG Swiss Corporation).The ABI will be calculated automatically for each foot by dividing the higher of the two systolic pressures in the ankle by the highest measurement of the two systolic pressures in the arm [35].

#### Cardiac assessment

The electrocardiographic examination will be performed with a General Electric MAC 3.500 ECG System (Niskayuna, New York, USA), that measures automatically the voltage and duration of waves and estimates the criteria of the Cornell voltage-duration product (Cornell VDP) to assess the LVH by the following equation: Men ((RaVL + SV3) * QRS) and women ((RaVL + SV3) * QRS + 6). LVH is defined as the voltage-duration product > 2,440 mm/ms [36]. We will estimate left ventricular mass index (LVMI) by Novacode equation [37].

#### Renal assessment

The kidney damage will be assessed by measuring creatinine plasma concentration, the glomerular filtration rate will be estimated by CKD-EPI (Chronic Kidney Disease Epidemiology Collaboration) [38] and the MDRD-IDMS (Modification of Diet in Renal Disease-Isotopic Dilution Mass Spectrometry) [39] equation and proteinuria will be assessed by the albumin/creatinine ratio following the 2007 European Society of Hypertension/European Society of Cardiology Guidelines criteria [40]. Subclinical organ damage will be defined as plasma creatinine between 1.3 - 1.5 mg/dl in men and 1.2 - 1.4 mg/dl in women, glomerular filtration rate below 60 ml/min or albumin/creatinine ratio > 22 mg/gr in men and 31 mg/gr in women. Renal disease will be defined as plasma creatinine of 1.5 mg/dl or higher in men and 1.4 mg/l in women or albumin/creatinine ratio > 300 mg/24h.

#### Evaluation of retinal involvement

Retinography will be performed with a Topcon TRC NW 200 non-mydriatic retinal camera (Topcon Europe B.C. Capelle a/d IJssel The Netherlands), obtaining images centered on the papilla, nasal and temporal. Once the images will be captured, two independent observers classified them according to the Keith-Wagener and Wong classification for hypertensive retinopathy [41,42], and serious international scale for diabetic retinopathy with a third reading being performed in cases where there were discrepancies. Grades III or IV (hemorrhages or exudates, papillary edema) are considered to be associated with cardiovascular disease [40].

As part of the project we include development of the second phase of software designed to semiautomatically measure the arterial (central retinal artery equivalent, CRAE) and venous caliber (central retinal vein equivalent, CRVE) of the retinal vascular tree and to automatically estimate the retina arteriovenous index, based on a method similar to that of the Atherosclerosis Risk in Communities (ARIC) study in the United States [43], making it possible to increase measurement precision and minimize interobserver variability. In this second phase we aim to automatize the software in order to avoid interobserver variability, and increase the efficiency of retinal evaluation so that the retinas are automatically evaluated by the developed tool and a final report is generated with the measurements made.

#### Validation of the retina software

For validation of the retina software in its semiautomated and automated versions, the following steps will be followed after prior training of the examiners who will evaluate the images obtained: 1.- Intraobserver variability: in order to assess the repeatability of the measurement, an operator must measure the same photograph in the same individual at least twice. To this effect, an operator will evaluate 200 images of a random sub-sample of 100 patients, with a difference of one week between the two evaluations. In this case the operator and the images analyzed will be the same on both days, and the operator will have no information on the previously performed evaluation. 2.- Interobserver variability: in order to assess the reproducibility of the measurement system, an operator different from that in phase 1 will evaluate the same 200 images previously analyzed. This operator will be blinded to the results already obtained in the previous phase. The two operators will have the same experience in the subject and in the use of the software, and both will receive the same prior training. 3.- Ocular variability: one month later, new photographs will be obtained of the same group of patients. These results will be evaluated by the operator of phase 1, and in this case the origin of the image, the patient characteristics and the results obtained in the previous evaluation will not be known. 4.- Furthermore, the retina software in its automated version (phase 2) generates the results relating to the retina arteriovenous index, CRAE and CRVE. In the semiautomated version the user participates and decides the interpretations made by the computer, and can change them if considered necessary. The automatic and semiautomatic evaluation of 200 images will be used to assess the degree of concordance between the two methodologies. In this way we will be able to show that the automatic method not only affords the same results but moreover also improves daily practice, affording greater objectivity and speed in producing the results. 5.- The validity of the measurement will be analyzed comparing the results with the carotid IMT as measure of vascular structure and with PWV as measure of vascular function and gold standard of arterial stiffness of the 300 patients included in the study. 6.- An analysis also will be made of the association of the estimated retina arteriovenous index and the evolution or appearance of new target organ damage and cardiovascular events during follow-up of the patients over the four years of the second phase of this project.

### Statistical analysis

The data will be presented with the mean and 95% confidence interval (95%CI) in the case of quantitative variables, and as frequency distributions for qualitative variables. The Pearson chi-squared test will be used to analyze associations between qualitative variables. Student's t test for independent samples will be used to compare the means of two groups, and analysis of variance (ANOVA) will be performed in the case of a larger number of groups. *Post hoc *contrasts will be made with the least significant difference (LSD) technique, with an alpha < 0.05. The relationship between quantitative variables will be analyzed using Pearson's correlation coefficient. Estimation of the slope of the straight line needed to calculate the ambulatory arterial stiffness index (AASI) will be made based on a linear regression model. Lastly, multiple linear regression and logistic regression analysis will be made to analyze the most significant variables conditioning the alterations in arterial pressure and vascular damage with the different methodologies used in the two groups. Hypothesis contrasting will establish an alpha risk factor of 0.05 as the limit of statistical significance.

Validation of the retina software: For evaluating the measurements of the retina arteriovenous index, CRAE and CRVE obtained in the three validation phases, calculation will be made of the intraclass correlation coefficient as comparison method, together with the Bland-Altman technique, which allows graphical evaluation of the concordance or agreement between the two measurement systems. The results obtained with the two types of measurements that can be made with the software (automated or semiautomated) will be analyzed with the intraclass correlation coefficient and Kappa concordance coefficient on categorizing the variable. This coefficient will allow us to evaluate the degree of concordance between the two types of results. The SPSS/PC+ version 18.0 statistical package will be used.

### Study limitations

The sample is composed of diabetics and non-diabetics, many of them with arterial hypertension referred by their primary care physician to the research unit for ABPM and the evaluation of cardiovascular risk. As a result, consecutive non-randomized sampling is involved. However, the large size of the sample partially offsets this limitation, and the real clinical practice conditions can bring us closer to the real-life situation than if more restrictive criteria were used for including the patients in the study. We will have diabetic, hypertensive and control subjects without these disorders for analysis and comparison of the results.

### Quality control

In order to ensure data quality, the nursing professionals in charge of data collection will receive specific training. Periodic external monitoring will be performed to verify adequate application of the methodology, both in performing the different examinations and in collecting the information.

### Ethical and legal issues

In order to guarantee data confidentiality, all the electronic and paper copies of the protocol, signed informed consent documents and results of the tests made in each of the patients will be kept locked in a safe place, and only the study investigators will have access to the data on the subjects who agree to participate in the study. The study has been approved (Agost 10, 2010) by the research ethics committee from University Hospital of Salamanca, Spain and complies with Spanish data protection law 15/1999 and its recently developed specifications (Royal Decree (RD) 1720/2007). Knowledge and agreement to cooperate has been established with the implicated services, signed by the legal representative of the centre. Written informed consent to participation in the study will be obtained in all cases.

## Discussion

The measurement of arterial pressure, both in the clinic and on an ambulatory basis, is very widespread in clinical practice, and its direct correlation to mortality and morbidity has been extensively studied [44-46]. However, the correlation of other parameters derived from ABPM and central arterial pressure with cardiovascular target organ damage, and the behavior of diabetic patients versus non-diabetic individuals, have not been fully clarified. With this study we hope to obtain information on the correlation of parameters such as the night/day arterial pressure ratio, AASI, symmetrical AASI, variability of arterial pressure and heart rate and other parameters to be defined with organ target damage - particularly vascular structure and function evaluated from the IMT and PWV - and endothelial dysfunction as assessed by biological parameters. Likewise, there are a series of parameters derived from pulse wave analysis (PWA) such as the central and peripheral augmentation index, whose role as predictors of cardiac or renal vascular damage and behavior in different groups of patients (e.g., diabetics versus non-diabetics) have not been fully clarified. In this sense, we hope to obtain sufficient information to clarify the role of these parameters.

We also hope to demonstrate the usefulness of the instrument developed for the automatic evaluation of retinal vascularization in the early detection of alterations in vascular structure and function and in the prognosis of middle-term cardiovascular morbidity. This tool could help improve the information derived from retinal photographs and make their use more efficient thanks to automatization of the procedures, in both diabetics and in individuals without diabetes. Lastly, we hope to clarify the prognostic role of the biological markers of endothelial dysfunction such as osteoprotegerin and endoglin, and to standardize their use in clinical practice.

## Abbreviations

AASI: Ambulatory arterial stiffness index; ABI: Ankle-brachial index; ABPM: Ambulatory blood pressure monitoring; AIx: Augmentation Index; ARIC: Atherosclerosis Risk in Communities study; ASCOT: Anglo-Scandinavian Cardiac Outcomes Trial; CAFE: Conduit Artery Function Evaluation; CKD-EPI: Chronic Kidney Disease Epidemiology Collaboration; CRAE: central retinal artery equivalent; CRVE: central retinal vein equivalent; DPB: Diastolic blood pressure; ESH: European Society of Hypertension; IMT: Intima-media thickness; MDRD-IDMS: Modification of Diet in Renal Disease-Isotopic Dilution Mass Spectrometry; OPG: Osteoprotegerina; PP: Pulse pressure; PWA: Pulse Wave Analysis; PWV: Pulse Wave Velocity; SBP: Systolic blood pressure.

## Competing interests

The authors declare that they have no competing interests.

## Authors' contributions

Conception of the idea for the study: LGO, ERD and MAGM. Development of the protocol, organization and funding: LGO, ERD, JIRR, CAC, CMS, MCPA, ERS and MAGM. Writing of the manuscript: LGO and MAGM. All the authors have read the draft critically, to make contributions, and have approved the final text. The project will be developed by Vaso risk group.

## Pre-publication history

The pre-publication history for this paper can be accessed here:

http://www.biomedcentral.com/1471-2458/11/266/prepub
